# Injectable Thermoresponsive Dual Nanocarrier Hydrogel for Local Tacrolimus Delivery with a Two-Phase Release Profile

**DOI:** 10.3390/pharmaceutics18060701

**Published:** 2026-06-07

**Authors:** Sanjida Ahmed Srishti, Paromita Paul Pinky, Diponkor Kumar Shill, Vidya Surti, Jelena M. Janjic

**Affiliations:** School of Pharmacy, Graduate School of Pharmaceutical Sciences, Duquesne University, Pittsburgh, PA 15282, USA

**Keywords:** tacrolimus, dual nanocarrier-loaded hydrogel, nanoemulsion, micelle, localized drug delivery

## Abstract

**Background:** Overexpression of immune cell populations leads to self-amplifying cytokine loops, contributing to chronic inflammation in both allograft rejection and autoimmune conditions. Tacrolimus (TAC), despite being a potent immunosuppressant, has limitations; its systemic adverse effects include nephrotoxicity, neurotoxicity, and high variability in tissue exposure in patients. Currently available therapeutic options are limited by the lack of targeted and localized drug delivery systems, resulting in ineffective control over drug-release behavior. Moreover, TAC being highly lipophilic poses challenges for formulation development. To address these gaps, this study focuses on developing a thermoresponsive hydrogel platform comprising distinct nanocarriers for localized delivery of TAC. The nanocarriers include nanoemulsion (NE) and micelles as TAC carriers, and their particle sizes are specifically engineered at the nanoscale for differential release behavior and to support immune cell targeting (macrophages and T-cells). Incorporation into a thermoresponsive hydrogel matrix enables it to act as a local depot at the injection site and deliver TAC with a slow, extended-release profile. **Methods:** TAC was loaded into a coconut-rich lipid-phase-based NE via high-pressure microfluidization. Simultaneously, TAC-loaded micelles were optimized using a full-factorial design of experiments (DoE) and manufactured via the thin-film hydration method. Both nanocarriers were evaluated for long-term colloidal stability assessments. Hydrogels were produced maintaining aseptic conditions for sterile batch production. Rheological characterization was performed to assess sol-gel transition, thermoreversibility, and injectability, and in vitro release studies were conducted to evaluate TAC diffusion from the developed nanoformulations. **Results:** Developed nanocarriers resulted in distinct particle sizes in NE (80–85 nm) and micelles (15–17 nm) with successful TAC loading maintaining long-term colloidal stability. The developed TAC-loaded dual-nanocarrier hydrogel (Dual-HG) showed thermoresponsive behavior and gelation at 37 °C, forming as a local depot. In vitro release studies showed slow and extended tacrolimus release from hydrogels and demonstrated particle size-dependent release behavior between the NE and micelle. **Conclusions:** Therefore, our study highlights a novel dual nanocarrier hydrogel platform combining TAC-NE and TAC-micelle for localized delivery. The findings support that nanocarriers can be engineered to modulate drug diffusion behavior. Notably, the dual nanocarrier within a thermoresponsive hydrogel platform can be used to deliver one or multiple drugs locally, minimizing systemic exposure when sustained local immunosuppression is required. The 25 mL scale sterile batch production of hydrogels emphasizes their suitability for future translational applications.

## 1. Introduction

Immune dysregulation is a central pathogenic driver of autoimmune, chronic inflammatory, and dermatological disorders, as well as allograft rejection [1]. The underlying basis behind persistent immune activation is complex and involves interrelated mechanism between the innate and adaptive immune systems [2]. Following internal or external stimuli, the key innate cells (e.g., macrophages, dendritic cells, neutrophils) become activated and contribute to the initiation of inflammation releasing pro-inflammatory cytokines such as TNF-α, IL-23, IL-6, and IL-1β [3,4,5]. The released cytokines further stimulate the activation of adaptive T-cell subsets (Th17, Th1, and Th22), leading to elevated secretion of pro-inflammatory cytokines including IL-17, IL-22, IFN-γ, and TNF-α [5,6]. Hence, dysregulation of these key innate immune cells and the adaptive T-cell subsets plays a major role in the interplay of cytokine networks [3]. Given that, the effector phase of immune dysregulation often occurs within the specific microenvironment of affected tissues or grafts [7]. As a result, local immunosuppression becomes critical for the treatment of inflammatory dermatological disorders and reducing graft rejection in vascularized composite allotransplantation (VCA), both of which have a substantial clinical burden [8,9]. Although the emergence of these conditions has distinct contexts, they share a common immunopathologic feature: sustained crosstalk between adaptive T-cell responses and innate immune activation via monocyte-derived macrophages [10,11,12]. Therefore, improved strategies for site-specific immunomodulation remain an ongoing clinical priority.

Among currently available immunosuppressive agents, tacrolimus (TAC; FK506; Prograf^®^), a U.S. FDA-approved macrolide immunosuppressant, is widely used in transplantation settings and dermatology for suppressing T-cell-mediated immune responses and downstream inflammatory signaling [10]. In transplant settings, TAC serves as a cornerstone immunosuppressive therapy by inhibiting alloantigen-driven T-cell activation, thereby reducing the risk of graft rejection [8]. In chronic inflammatory skin conditions such as psoriasis, TAC can also modulate cytokine signaling by T-cell suppression and reducing Th1- and Th17-associated inflammatory pathways [13]. Mechanistically, tacrolimus binds intracellular FK506-binding protein 12 (FKBP12), forming a complex that inhibits calcineurin phosphatase activity and prevents activation of nuclear factor of activated T cells (NFAT). This suppresses transcription of interleukin (IL)-2 and other T-cell growth factors, thereby limiting T-cell proliferation and effector function across disease contexts. In addition to calcineurin inhibition, TAC has been shown to modulate mitogen-activated protein kinase (MAPK) signaling pathways in T cells, further attenuating inflammatory cytokine production [14,15,16]. Although TAC is known to act on T-cell suppression, calcineurin and MAPK signaling pathways are also present in monocyte-derived macrophages. Emerging evidence suggests that TAC exerts immunomodulatory effects beyond adaptive immunity [17,18]. TAC modulates innate immunological responses such macrophage phenotypes and inflammatory cytokine production, suggesting immunoregulatory potential beyond adaptive immune suppression [17,19]. The capacity of TAC to influence both calcineurin and MAPK-dependent pathways across different immune cell types indicates that enhancing drug delivery to both adaptive and innate immune systems could potentially enhance treatment effectiveness. However, TAC presents significant formulation and pharmacokinetic challenges that limit its therapeutic potential. Because of its high lipophilicity, poor aqueous solubility, and instability, TAC exhibits limited tissue penetration and variable absorption. Oral administration is further complicated by extensive first-pass metabolism in the intestine and liver, resulting in an approximate bioavailability of 21% and substantial inter- and intraindividual variability in drug exposure [20,21]. Consequently, systemic delivery often produces broad immunosuppression without achieving optimal drug concentrations at target tissues and requires frequent dosing. These limitations underscore the need for delivery systems capable of improving tacrolimus stability, tissue retention, and localized immunomodulation.

Nanoformulations as delivery platforms have shown promise in addressing these limitations [22,23,24,25]. Specially, nanoemulsions (NEs) are kinetically stable colloidal systems composed of oil, surfactants and water, in which lipophilic drugs can be incorporated into the internal oil phase and dispersed in an external aqueous phase as nanosized droplets [26]. Moreover, NEs can be engineered to have specific particle size and formation of the nanodroplets utilize high-energy techniques like microfluidization, sonication, homogenization [27,28]. NEs with particle sizes in the 80–100 nm range are suitable for phagocytic macrophage uptake [29,30]. Another nanocarrier approach is micelles which are nanosized self-assembled colloidal carriers formed by amphiphilic molecules in an aqueous phase, with a hydrophobic core capable of incorporating lipophilic drugs and a hydrophilic outer shell that stabilizes the system in water [31,32]. The small particle size may facilitate faster diffusion within T-cell-driven inflammatory microenvironments [33,34]. However, current TAC formulations are primarily designed for systemic administration rather than localized drug delivery and lack site-specific targeting capability [35]. As a result, there is a need for drug delivery systems that enable localized and targeted TAC delivery with prolonged tissue retention. Pluronic-based systems are promising in this regard, because of the thermoresponsive behavior which allows localized depot formation at the administration site [17].

Our previous work reported thermoresponsive TAC-loaded nanoemulgel (TAC-NEG), a single nanocarrier-based platform prepared by incorporating TAC-loaded NE (TAC-NE) into a Pluronic-based hydrogel matrix, which modulated macrophage inflammatory activity in RAW 264.7 cells through reductions in nitric oxide, IL-6 and TNF-α production [17]. Thus, it highlighted the promise of TAC nanoformulations and provided a basis for exploring dual nanocarrier strategies within localized hydrogel depots. Additionally, other studies have also reported on the development of single nanocarriers, such as NEGs [36], liposomal gels [37,38], nanoparticle [39] or nanovesicular carriers [40], etc., for localized TAC delivery. However, these systems are primarily based on single nanocarrier-based release contributions and are not designed for independently modulating early and sustained drug release within one depot formulation.

To address this therapeutic need, we engineered an injectable dual-nanocarrier-loaded hydrogel platform designed to simultaneously deliver TAC to immune cell populations involved in dysregulation and understand how distinct nanocarriers may influence drug diffusion from the carrier. The relatively faster TAC release from the smaller micelles and the slower release from the nanoemulsion may allow a two-phase local delivery profile with earlier TAC availability followed by sustained drug exposure over time targeting the key immune cells for local immunomodulation. The development of the TAC-NE was guided by prior experience in tacrolimus nanoformulation design [17,28,41]. Given the physicochemical sensitivity of TAC [42], the importance of colloidal storage stability was emphasized for ensuring formulation robustness. A QbD-guided design of experiments (DoE) approach was adopted to optimize the TAC-micelle formulation, in accordance with ICH Q8 principles [43]. The systematic strategy facilitates understanding of how formulation factors affect critical response attributes and enables formulation optimization.

To the best of our knowledge, this work introduces for the first time an injectable thermoresponsive dual-nanocarrier hydrogel platform for localized tacrolimus delivery, incorporating the optimized TAC-NE and micelles within a single depot-forming system. The study describes the development and optimization of both nanocarriers, their incorporation into the hydrogel matrix, and the evaluation of long-term colloidal stability, rheological behavior, and in vitro release characteristics. The findings of this study demonstrate distinct particle size-dependent release behavior and support the potential of this sterile hydrogel platform for prolonged local tacrolimus retention while minimizing systemic exposure. The dual nanocarrier strategy for TAC delivery may offer advantages in improving local immunomodulatory performance by combining early and prolonged TAC availability within one depot formulation, thereby establishing a strong formulation foundation for future biological validation.

## 2. Materials and Methods

### 2.1. Materials

Tacrolimus-FK506 was obtained from Selleckchem, USA (Houston, TX, USA). Coconut oil was obtained from MP, Biomedical (Solon, OH, USA), and dimethyl sulfoxide (DMSO) was purchased from Sigma-Aldrich (St. Louis, MO, USA). Pluronic^®^ F-127 and Pluronic^®^ P105 were purchased from Sigma-Aldrich (St. Louis, MO, USA) and US Biological Life Sciences (Salem, MA, USA), respectively. Kolliphor^®^ EL (Cremophor EL; CrEL) was obtained from Spectrum (New Brunswick, NJ, USA). Indocyanine green (ICG) was purchased from Sigma-Aldrich (St. Louis, MO, USA). Octadecyl amine (Sterile Amine; SA) and 2-(2-Ethoxyethoxy) ethanol (Transcutol) were purchased from Sigma-Aldrich (St. Louis, MO, USA) and Spectrum (New Brunswick, NJ, USA), respectively. Dulbecco’s Modified Eagle Medium (DMEM) was purchased from Fisher Scientific (Hampton, NH, USA) and fetal bovine serum (FBS) was sourced from ATCC (Manassas, VA, USA). DMEM without phenol red (1×, ref. 21041-025) was obtained from Gibco, Thermo Fisher Scientific (Waltham, MA, USA). The Raw 264.7 macrophage cell line was obtained from ATCC (Manassas, VA, USA). The CellTiter-Glo^®^ 2.0 luminescent cell viability assay was purchased from Promega Corporation (Madison, WI, USA). Sterile 1× Dulbecco’s phosphate-buffered saline (DPBS) and Tween^®^ 80 were purchased from Sigma-Aldrich (St. Louis, MO, USA). The LM20 microfluidizer^®^ from the Microfluidics Corporation (Westwood, MA, USA) was used for manufacturing nanoemulsion. Solvents used for high-performance liquid chromatography (HPLC) including acetonitrile HPLC grade and phosphoric acid were obtained from Thermo Fisher Scientific (Waltham, MA, USA) and Sigma-Aldrich (St. Louis, MO, USA), respectively. The organic solvent acetone was purchased from Sigma-Aldrich (St. Louis, MO, USA).

### 2.2. Methods

#### 2.2.1. Preparation of Tacrolimus-Loaded Nanoemulsion (TAC-NE) and Drug-Free ICG Nanoemulsion (ICG DF-NE)

Nanoemulsions (TAC-NE and ICG DF-NE) were prepared using a high-pressure microfluidization technique utilizing an LM20 microfluidizer^®^. First, the coconut oil, Pluronic P105 and Cremophor EL were heated at high temperature (45–51 °C) for 60 s, transitioning into homogenous liquid phase for uniform mixing [28,41]. The nanoformulation consisted of 23% (*w*/*v*) coconut oil, 7.5% (*w*/*v*) Cremophor EL, and 3.33% (*w*/*v*) Pluronic P105. Tacrolimus was then incorporated at concentrations of 0.5% or 0.2% (*w*/*w*), along with 1.66% (*w*/*v*) DMSO as a co-solvent. The mixture was set to pre-mix for 3 h for complete solubilization of tacrolimus in the non-aqueous phase while maintaining the temperature between 35 and45 °C to avoid any solidification of coconut oil. Subsequently, the pre-mix was homogenized, while the aqueous phase DPBS was gradually added to form the coarse pre-emulsion. The LM20 microfluidizer was primed with DPBS and the resulting pre-emulsion was then processed using the LM20 microfluidizer at 20,000 PSI in 3 passes, forming NE droplets. To prepare DF-ICG NE, the ICG-SA ionic complex was prepared by dissolving in a solvent mixture of DMSO and Transcutol^®^ (1:1, *v*/*v*), followed by overnight mixing [28,44]. The ICG stock was incorporated into the pre-mix 30 min prior to processing to minimize prolonged thermal exposure. The TAC-NE batches and DF-ICG NE batches were prepared using identical processing parameters to ensure consistency between formulations.

#### 2.2.2. Preparation of Tacrolimus-Loaded and Drug-Free Micelles

Tacrolimus-loaded micelles (TAC-micelles) were produced following an optimized thin-film hydration method using a rotary evaporator [45]. At first, the Pluronic P105 (2.4 g) and Cremphor EL (1.2 g) were dissolved in 10 mL of acetone individually and overnight pre-mixing was done for complete solubilization. A total of 4 mg of TAC was dissolved in 1 mL of acetone. Subsequently, 2 mL of P105 (1.61 g *w*/*v*) and 2 mL of CrEL (1.68 g *w*/*v*) were combined with dissolved TAC in a 250 mL round-bottomed flask and mixed for 30 min at 150 rpm, maintaining the temperature at 37 °C. Acetone was evaporated slowly at high vacuum (556 mbar) to form a thin film and the final thin film layer was hydrated with 8 mL of distilled water to a final TAC concentration of 0.5 mg/mL (0.005% *w*/*w*). The optimized micelle formulation has a final concentration of 6% P105 and 3% CrEL in the micelle. In addition to the optimized TAC-micelle, eight more batches were formulated following the same procedure and varying the final percentage of P105 and Cremophor EL according to the experimental design in Table 1. Drug-free (DF)-micelles were prepared following the same procedure using only 2 mL of each surfactant dissolved in acetone without TAC.

#### 2.2.3. Preparation of Single-Nanocarrier and Dual-Nanocarrier-Loaded Hydrogels

For manufacturing the hydrogels, at first 30% (*w*/*w*) Pluronic-F127 was prepared in 1× PBS (Phosphate buffered saline) and autoclaved to maintain aseptic conditions. For sterile hydrogel preparation, all components of the planetary centrifugal mixer (THINKY mixer) including the cup-holder were sterilized with 70% isopropyl alcohol (IPA) prior to use. The cup-holder utilized for holding the gels was kept under UV light for sterilization. For producing the TAC-NEG, the single-nanocarrier-loaded hydrogel, 15 g of F-127 and 10 g of TAC-NE were added to the cup and rotated at 2000 rpm for 90 s in the THINKY mixer [17,45,46]. The high centrifugal force disperses the NE into the Pluronic F-127-based hydrogel matrix. Following a similar approach, the TAC-micelle formulation (2.5 g) and TAC-NE (7.5 g) and Pluronic F-127 (15 g, *w*/*v*) were added into the THINKY cup and mixed in the planetary centrifugal mixer following the same processing parameters to form the sterile TAC-loaded dual hydrogel (TAC-Dual HG). The drug-free dual hydrogel (DF-Dual HG) was also produced using the ICG-labeled DF-NE and DF-micelle following a similar formulation and manufacturing approach. The final concentration of F-127 in the TAC-NEG, TAC-Dual HG and DF-Dual HG was 18% (*w*/*v*). Throughout the manufacturing process, the Pluronic solution, mechanical pipettes and cup-holder were kept on ice to avoid any gelation.

#### 2.2.4. Tacrolimus Solubility Study

The TAC solubility was evaluated on individual formulation components included in the non-aqueous phase of the NE and micelle. In total, 1 g of coconut oil, Pluronic P105, CrEL and DMSO were added in amber vials. The coconut oil and P105 were melted and the temperature was below 40 °C. After that, in each ingredient 50 mg of TAC was incorporated and pre-mixed with magnetic stirring for 3 h at 37–40 °C. The temperature was set at an elevated level to account for manufacturing conditions. After 3 h of pre-mixing, samples were diluted with pure acetonitrile. Then, 1 mL of primary diluted solution was collected and centrifuged at 5000 rpm. The supernatant was collected and secondary dilution was performed in the mobile phase containing acetonitrile: water: phosphoric acid (70:30:0.05). Samples were prepared at a final concentration of 100 µg/mL in triplicate (*n* = 3), and tacrolimus concentration was determined from the area under the curve (AUC) using the optimized reverse phase high-performance liquid chromatography (RP-HPLC) method described in Section 2.2.5.

#### 2.2.5. Reverse Phase High-Performance Liquid Chromatography (RP-HPLC)

The TAC loading efficiency in the developed formulations were calculated using an optimized method of RP-HPLC on a Thermo Scientific Vanquish HPLC system (Thermo Fisher Scientific, Waltham, MA, USA) [17]. For chromatographic separation, a C18 column (Phenomenex, Torrance, CA, USA) with a mobile phase containing acetonitrile: water: phosphoric acid (70:30:0.05) was utilized. The HPLC was operated using a flow rate of 0.75 mL/min, column temperature of 60 °C and UV channel setting at 210 nm with an injection volume of 50 µL. The method parameters were adopted from the optimized protocol [17,41]. Following that, a calibration curve was prepared using a standard TAC solution prepared in mobile phase with a concentration ranging from 1 µg to 250 µg (*n* = 3). The calibration curve was generated with the determination of the limit of detection (LOD) and limit of quantification (LOQ). To determine the TAC concentration in TAC-NE (0.45 µm filtered), TAC-micelle (0.22 µm filtered) and TAC-HGs, samples were initially diluted in acetonitrile. Primary samples with TAC-NE and TAC-HGs were centrifuged at 5000 rpm. The resulting supernatant was collected and diluted into the mobile phase. The final concentrations were 100 µg/mL for TAC-NE samples, 20 µg/mL for TAC-micelles and 10 µg/mL for TAC-NEG and TAC-dual HG samples. TAC concentration was quantified based on the AUC using the calibration curve. The percentage loading efficiency was calculated as the ratio of the amount of TAC present in the formulation to the initial amount of TAC used during preparation, expressed as a percentage.

#### 2.2.6. Design of Experiment (DoE)

A three-level, two-factor full-factorial DoE was applied for optimizing the TAC-micelle formulation. The independent factors were the concentration of P105 (2%, 4%, and 6%) and CrEL (3%, 6%, and 9%) in the final micelle formulation. The response variables were the particle size, distribution type, poly dispersity index (PDI), and % drug loading efficiency from Day 1 to Day 30. The JMP^®^ software (version 17.2.0, SAS Institute Inc., Cary, NC, USA) was utilized for setting factor levels and generated 9 experimental runs (Table 1).

#### 2.2.7. Particle Size and Poly Dispersity Index (PDI) Analysis

The particle size and PDI of the nanoemulsions, micelles and hydrogels were determined using dynamic light scattering (DLS). Measurements were performed using a Zetasizer Nano ZS (Malvern Instruments, Worcestershire, UK) with a backscatter angle of 173 degrees and temperature of 25 °C, and the nanoemulsion and hydrogels were diluted at a ratio of 1:40 (*v*/*v*) and micelle were diluted at 1:5 (*v*/*v*) ratio with deionized water. Values were reported as the mean ± standard deviation for triplicate measurements.

#### 2.2.8. Near-Infrared Fluorescence (NIRF) Analysis of NEs and HGs

The NIRF imaging was completed on the Li-COR Odyssey M instrument (LI-COR Biosciences, Lincoln, NE, USA). The ICG-NE and ICG Dual HG were serially diluted with deionized water starting from 1:5 to 1:160 dilution [47]. Each of the measuring concentrations was then transferred to a 96-well plate at 100 µL/well in triplicate. Fluorescence signal intensity was measured using an Odyssey M imager (LI-COR Inc., Lincoln, NE, USA) at 800 nm wavelength with a 3.75 focus offset setting. Quantification of fluorescence signal intensity was performed using Image studio software (version 5.2; LI-COR Biosciences, Lincoln, NE, USA).

#### 2.2.9. Physical Stress Testing of NEs: Centrifugation and Filtration

The NEs were subjected to centrifugation at 1100 rpm and 3000 rpm for 5 min at ambient temperature. The filtration test was done by passing the NEs through a 0.45 μm filter (Merck Millipore Ltd., Burlington, MA, USA). The particle size and PDI values were measured at 1:40 (*v*/*v*) dilution with deionized water followed by centrifugation and filtration.

#### 2.2.10. Stability Testing in Biological Medium of NEs

A stability study in biological medium was conducted by exposing the NEs (0.45 µm filtered) to three different conditions including water, DMEM (Dulbecco’s Modified Eagle Medium), and 20% FBS (fetal bovine serum) in DMEM. The NEs were placed in a 1.5 mL Eppendorf tube at 1:40 (*v*/*v*) dilution with these three media. Samples were incubated at 37 °C for 72 h, and particle size measured before and after incubation using DLS.

#### 2.2.11. Rheological Characterization

A comprehensive rheological study was performed on hydrogels (TAC-NEG, TAC-Dual HG batches) to assess the rheological behavior utilizing a Discovery HR-20 rheometer (TA Instruments, Waters™, Milford, MA, USA). A 20 mm parallel plate stainless steel geometry and a 1 mm gap were utilized for this study. The hydrogels were kept on ice for at least 30 min prior to loading into the plate. The Peltier plate temperature was equilibrated to 10 °C prior to each run to avoid any gelation during sample loading. A total of 360 µL of sample was loaded for each measurement. The oscillatory amplitude sweep measurements were performed at 37 °C with varying strain from 0.1% to 100% at a constant angular frequency of 10 rad/s to determine the linear viscoelastic region, storage modulus (G′) and loss modulus (G′′). Frequency sweep studies were conducted at 37 °C using a constant strain of 0.1%, with angular frequency ranging from 0.1 to 100 rad/s. The temperature ramp studies were performed from 5 to 50 °C with a temperature rate of 5 °C/min and shear rate of 100/s to confirm the sol-to-gel transition of the hydrogels. The samples were loaded and measured in triplicate (*n* = 3). For further assessments, the storage modulus (G′) and loss modulus (G′′) were evaluated with an increase in temperature from 10 °C to 50 °C to assess the gelation starting point. Additionally, rheological studies including on the alternate step strain (0.1% and 100%) and alternate step temperature (10 °C or 37 °C) were conducted to assess the injectable and thermoreversible characteristics. Flow sweep studies were conducted at 25 °C by varying the shear rate from 0.1 to 100 s^−1^ to evaluate viscosity changes under different shear conditions.

#### 2.2.12. Dissolution Test to Evaluate the Release of Indocyanine Green (ICG)-Labeled Nanoemulsion Droplets from Dual Hydrogel

The study was performed by measuring 3 mL of ICG-DF DUAL HG in a 50 mL falcon tube and placing it in the incubator at 37 °C for 5–10 min for complete gel formation. Then, 12 mL of 10% FBS in DMEM was added into the tube without disturbing the hydrogel. The tube was always kept covered with aluminum foil and capped tightly. At each sampling time (0, 1, 3, 6, 24, 30, 48, 54, and 72 h), 100 μL of the medium was taken in triplicate from different locations and transferred to a 96-well plate without interrupting the hydrogel. Finally, fluorescence signal intensity was measured at 800 nm wavelength using the Li-COR Odyssey M with a 3.75 focus offset. To maintain the medium volume in the tube, 300 μL of fresh medium was added to maintain a constant release volume after each sampling point.

#### 2.2.13. In Vitro Tacrolimus Release Test (IVRT)

In vitro tacrolimus release studies were performed using a vertical Franz cell diffusion system (Conduct Science, Skokie, IL, USA) to evaluate the release and diffusion behavior of TAC from the developed formulations [48,49]. A regenerated cellulose membrane (Ultracel^®^ 30 kDa molecular weight cut-off (MWCO), 25 mm diameter; MilliporeSigma, Burlington, MA, USA) was placed between the donor and receptor compartments. 1× PBScontaining 0.1% (*v*/*v*) Tween 80 was used as release media adapted from [17,41] and 15 mL of the release media was placed in the receptor compartment. Sink conditions were maintained with continuous stirring at 350 rpm and temperature was maintained at 37 ± 0.5 °C. The quantified TAC-loaded formulations including TAC-NE, TAC-micelle, TAC-NEG, TAC-Dual HG were placed in the donor compartment in triplicate (*n* = 3). A volume of 1 mL of sample for TAC-NE, TAC-NEG, TAC-Dual HG and 1.5 mL of TAC-micelle sample was loaded into the donor compartment. Additional 0.5 mL of TAC-micelle was considered due to its liquid consistency and lower TAC concentration (0.36 mg/mL) in comparison with other nanoformulation groups, allowing uniform distribution and ensuring sufficient drug loading in the donor compartment for reliable TAC quantification. At predefined time intervals (1, 3, 6, 24, 48, 72, 120, and 168 h), 1 mL samples were withdrawn using a sterile syringe from the receptor compartment and immediately replaced with an equal volume of fresh release medium to maintain constant volume sink conditions. The samples were collected in triplicate and analyzed using the RP-HPLC method described in Section 2.2.5 and the data were presented as the percentage of loaded tacrolimus dose for each formulation group.

#### 2.2.14. Sterility Testing

Following the USP 71 guideline, sterility assessment was adopted following previously published studies [45,47,50] and performed to evaluate the sterility profile of multiple TAC-NEG batches. This was a preliminary assessment confirming the sterile profile of the TAC-NEGs. Thioglycolate and trypcase soy broth (MilliporeSigma, Burlington, MA, USA) were used to assess microbial contamination under anaerobic and aerobic conditions, respectively. For each sample from an independent batch, the formulation was diluted in a 1:80 ratio (*v*/*v*) in each medium (thioglycolate or trypcase soy broth) by adding 375 µL of sample to 29.625 mL of sterile medium and mixed thoroughly. Then, 10 mL of aliquots was transferred into sterile 15 mL falcon tubes and control samples with only fresh medium were also prepared. All thioglycolate samples were incubated at 37 °C and trypcase soy samples were incubated at 25 °C. These samples were monitored over a 30-day period (Day 0, Day 14, and Day 30). Sterility was initially assessed by visual inspection for turbidity. The pH of all samples was measured using a calibrated pH meter (Orion Star A111, Thermo Fisher Scientific, Waltham, MA, USA). Calibration of the pH meter was performed prior to measurements using standard buffer solutions (pH 4.0, 7.0, and 10.0). The pH probe was rinsed with deionized water between measurements, and readings were recorded once stabilized. All measurements were performed in triplicate, and results are reported as the mean ± standard deviation. Sterility was confirmed by the absence of visible turbidity and no significant changes in pH over time compared to Day 0.

#### 2.2.15. Cell Culture and Viability Assay

The cell viability of TAC-NE, TAC-micelle, Free TAC, DF-Dual HG, TAC-NEG and TAC-Dual HG was assessed using the RAW 264.7 macrophage cell line (ATCC TIB 71) following a previously published protocol [17,51]. Briefly, RAW 264.7 macrophages were seeded at 10,000 cells per well in a 96-well plate followed by 24 h of incubation at 37 °C with 5% CO_2_ in a humidified incubator. Post-incubation, cells were treated with TAC-NE diluted with full cell culture medium at different TAC concentrations (3.125 µM–400 µM), either for 6 h or 24 h in a humidified incubator at 37 °C with 5% CO_2_. Free TAC, solubilized in DMSO containing an equivalent concentration of TAC-NE, was used as a free drug control. The required dilution of free TAC was prepared using full cell culture medium. For TAC-micelle, drug-free dual hydrogel, TAC-NEG and TAC-Dual Hydrogel, the required treatment concentrations (ranging from 0.625 to 80 μL/mL) were prepared using full cell culture medium. Subsequently, cells were treated with these treatment groups for 24 h in a humidified incubator at 37 °C with 5% CO_2_. The cell viability was measured using the Luminescent Cell Titer-Glo 2.0^®^ assay kit (Promega, Madison, WI, USA). After completion of the required treatment period, the treatment solutions in each well were replaced with 100 µL of fresh cell culture medium and 40 µL of Cell Titer-Glo reagent. The 96-well plate was then shaken at 50 rpm for 15 min using a plate shaker. Finally, the cell lysates were transferred from the culture plate to an opaque white 96-well plate and luminescence was measured using a BioTek Synergy HTX Multi-Mode plate reader (Agilent, Santa Clara, CA, USA).

#### 2.2.16. Statistical Analysis

The statistical analysis was performed utilizing JMP^®^ software (version 17.2.0 and Graph Pad prism version 10 (GraphPad Software, Boston, MA, USA). Multiple linear regression (MLR) was performed using JMP^®^ software to evaluate the effects of independent variables and their interactions with the response variables. Leverage plots were used to assess the significance and contribution of individual factors and interaction terms. Comparisons between formulation groups on particle size, %loading efficiency and %cumulative release, etc., were performed using either a two-tailed unpaired *t*-test or one-way and two-way analysis of variance (ANOVA), as appropriate. A *p*-value of <0.05 was considered as statistically significant.

## 3. Results

### 3.1. Optimization of Nanoemulsion for High-Dose Tacrolimus Encapsulation: Preparation and Characterization

**Figure 1:**
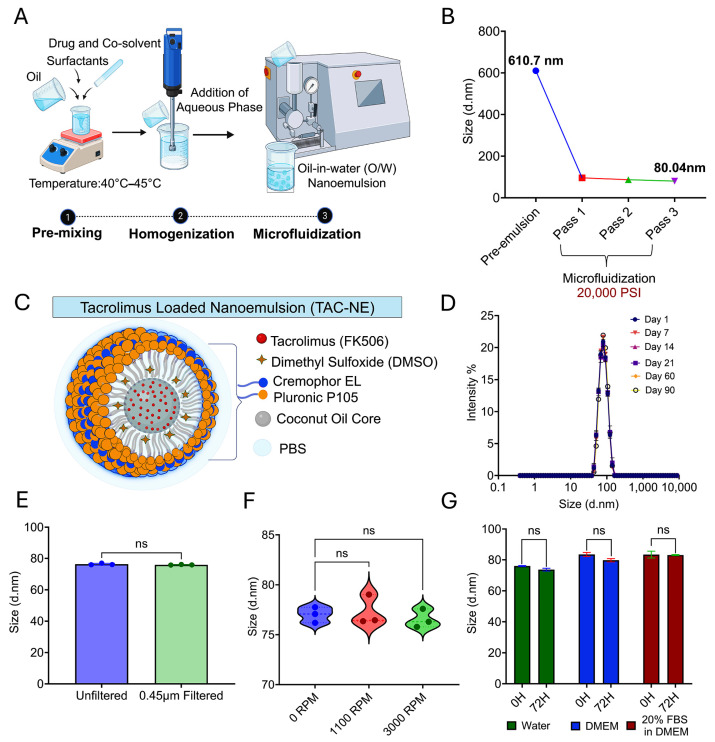
Manufacturing procedure, structural illustration, and stability assessment of TAC-NE. (**A**) Schematic illustration of three-step nanoemulsion manufacturing process. (**B**) Particle size reduction in each pass during microfluidization. (**C**) The structure of tacrolimus-loaded oil-in-water nanoemulsion having the hydrophobic drug tacrolimus in the core oil droplet stabilized by the surfactants in the interface and surrounded by the aqueous phase. (**D**) The assessment of particle size distribution until day 90. (**E**) The impact of filtration on TAC-NE was determined by comparing the particle size of unfiltered and filtered (0.45 µm filter) TAC-NE. (**F**) The impact of centrifugation on TAC-NE was determined by measuring the particle size of NE upon exposure with different centrifugation speed. (**G**) The impact of serum was studied by incubating TAC-NE in biological media and water at 37 °C for 72 h. The data represents the average ± SD (*n* = 3), with ns representing non-significant. The illustrations were Created in BioRender. Janjic, J. (2026) https://BioRender.com/ligfc9n.

Our lab previously developed TAC-loaded nanoformulations [17,41] and confirmed successful uptake by RAW 264.7 macrophages during in vitro studies [17]. In this study, we made further efforts to improve TAC loading in nanoemulsion with a high dose of TAC (0.5% *w*/*v*) for efficient macrophage uptake and therapeutic efficacy. To enhance the encapsulation of TAC, coconut oil as a saturated fatty acid-rich lipid, amphiphilic surfactants (Cremophor EL and Pluornic P105^®^) and PBS as the aqueous phase were used for producing nanoemulsion using the LM20^®^ microfluidizer. Figure 1A represents the three manufacturing steps for producing nanoemulsion. An increased temperature (40 °C to 45 °C) above the coconut oil melting point was maintained throughout the manufacturing steps following the pre-mixing and homogenization with the aqueous phase. A concentration of 5000 µg/mL of FK-506 tacrolimus was incorporated during pre-mixing. The pre-emulsion was processed in the LM20 microfluidizer with three passes at 20,000 PSI. Figure 1B shows the particle size of the pre-emulsion after homogenization along with a schematic representation of particle size reduction during each pass. The final particle size after the third pass was approximately 80.04 nm from 610.7 nm. The particle size was monitored in real time as part of the manufacturing screening process to determine whether additional processing steps were required. Figure 1C shows the illustration of the structure of nanoemulsion where the highly lipophilic tacrolimus is incorporated within the coconut oil core, while the hydrophobic regions of the surfactants associate with the core and the hydrophilic regions orient toward the aqueous phase. The long-term colloidal stability of nanoemulsion was evaluated based on particle size. Figure 1D demonstrates a uniform size distribution from Day 0 to Day 90. This result confirms that TAC-NE maintains its stability over an extended period, supporting its potential for longer shelf life. Furthermore, TAC-NE was filtered using 0.45 µm filter to ensure sterility and the particle size was assessed to evaluate the impact of filtration. Figure 1E shows there is no significant impact on particle size observed between the unfiltered and filtered nanoemulsion samples. Figure 1F shows that centrifugation at 1100 rpm and 3000 rpm did not significantly affect particle size compared to the 0 rpm (control) as confirmed by a two-way ANOVA, indicating no measurable difference in particle size. The TAC-NE was further exposed to biological media including DMEM and 20% FBS in DMEM and water (control) to assess the impact on particle size and Figure 1G shows no significant change between 0 and 72 h of incubation confirmed by separate *t*-tests performed for each group.

**Figure 2:**
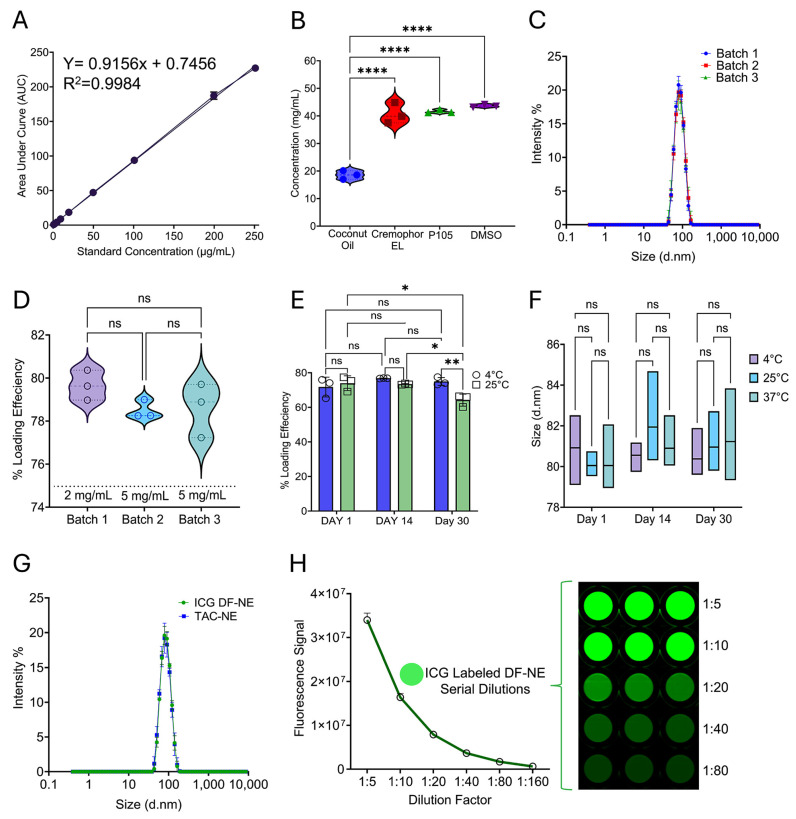
Characterization of tacrolimus loading, optimization of storage conditions of nanocarrier and comparison of ICG-labeled DFNE and TAC-NE. (**A**) Calibration curve for tacrolimus quantification established using RP-HPLC with UV detection at 210 nm. (**B**) Solubility screening of individual formulation components involved in optimized nanoformulation. (**C**) Particle size distribution of independently prepared replicate batches measured by dynamic light scattering (DLS). (**D**) Drug loading efficiency (%) of three independent batches of TAC-NE. (**E**) Effect of storage temperature on drug loading efficiency, evaluating formulation stability under different thermal conditions. (**F**) Effect of storage temperature on droplet size of TAC-NE, assessed to determine physical stability. (**G**) Comparative fluorescence distribution of ICG-labeled DF-NE and TAC-NE. (**H**) Fluorescence intensity of ICG-labeled nanoemulsion at varying dilution factors, indicating signal stability and detectability. Data are presented as the mean ± standard deviation (SD) (*n* = 3). Two-way ANOVA followed by Tukey’s multiple comparisons test was done, with ns representing non-significant, (*p* > 0.05), * (*p* < 0.05), ** (*p* < 0.01), **** *p* < 0.0001.

To quantify the loaded TAC concentration in the nanoemulsion, the calibration curve for TAC quantification was established using the optimized RP-HPLC method [17] with UV detection at 210 nm, operated at a flow rate of 0.75 mL/min and using a C18 column temperature of 60 °C. Figure 2A shows the calibration curve was constructed over a concentration range of 1 µg to 250 µg, which included the TAC-NE sample concentration of 100 µg used for HPLC quantification. The replicates of samples showed low variability and excellent linearity with a regression equation of y = 0.9156x + 0.7456. The limit of detection (LOD) and limit of quantification (LOQ) were determined to be 7.44 µg/mL and 22.53 µg/mL, respectively, indicating adequate sensitivity for quantifying tacrolimus. A solubility study of TAC was conducted for each ingredient in nanoemulsion when 50 mg of TAC was dissolved in 1 g of each ingredient. Figure 2B shows that compared to coconut oil, both surfactants and DMSO demonstrated a significantly higher solubility profile of tacrolimus (*p* < 0.0001) as confirmed by a two-way ANOVA. The NE carrier described in Figure 1 was further manufactured into three new batches for assessing reproducibility. Batch 1 was manufactured (0.2% *w*/*v* tacrolimus) and Batches 2 and 3 were manufactured with 0.5% TAC, indicating the dosing flexibility of this nanocarrier. Figure 2C shows comparable and overlapping particle size distributions among the three batches. The percentage of loading efficiency was evaluated using an optimized RP-HPLC method, and based on the AUC the % loading efficiency was calculated using the following equation,

(1) $$\%\mathrm{Loading}\mathrm{Efficiency}\mathrm{is}\mathrm{calculated}\mathrm{as}\frac{\mathrm{Weight}\mathrm{of}\mathrm{drug}\mathrm{in}\mathrm{product}}{\mathrm{Weight}\mathrm{of}\mathrm{drug}\mathrm{used}\mathrm{to}\mathrm{make}\mathrm{product}}\times 100$$

Across the three independent batches, the average TAC loading was 79.3% ± 0.6, indicating high loading efficiency and consistent reproducibility as shown in Figure 2D. Moreover, the stability of tacrolimus loading in the nanoemulsion under different storage conditions (4 °C and 25 ± 2 °C) was evaluated from Day 1 to Day 30, as shown in Figure 2E. No significant changes in loading efficiency were observed from Day 1 to Day 14 at either 4 °C or 25 °C. However, after one month of storage at room temperature 25 ± 2 °C, the TAC loading was significantly reduced (*p* < 0.05) compared to Day 14 and the 4 °C storage condition group on Day 30 (*p* < 0.01), determined by two-way ANOVA followed by Tukey’s multiple comparisons test. The TAC loading of the nanoemulsion sample stored at 4 °C was stable with no significant changes until Day 30. Figure 2F shows the evaluation of colloidal stability at different temperatures and demonstrated no significant differences in particle size across the evaluated storage temperatures (4 °C, 25 ± 2 °C, and 37 °C) (*p* > 0.05). Following that, DF-NE was prepared as a control formulation using the same formulation component. Additionally, an ICG-conjugated fluorescent dye was incorporated into the nanoemulsion to evaluate its impact on TAC loading in the nanocarrier. As shown in Figure 2G, the particle size distribution profiles were similar for both DF-NE and TAC-NE, indicating that TAC incorporation did not significantly affect the colloidal characteristics of the system. Figure 2H shows that the nanoemulsion system was able to successfully incorporate ICG as a fluorescent dye and the serial dilution showed a concentration-dependent decrease in fluorescence intensity, confirming successful incorporation and detectability of ICG within the nanoemulsion.

### 3.2. Preparation of Sterile Tacrolimus-Loaded Nanoemulgel (TAC-NEG): A Single-Nanocarrier-Loaded Hydrogel

**Figure 3:**
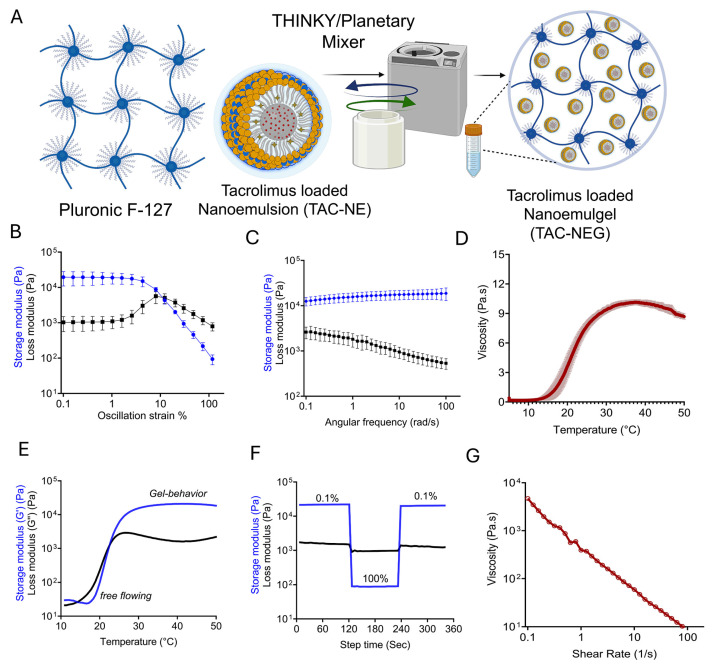
Preparation and rheological characterization of TAC-NEG. (**A**) Schematic illustration of TAC-NEG preparation via incorporation of TAC-NE into thermoresponsive hydrogel. (**B**) Oscillation amplitude sweep test at 0.1% to 100% oscillation strain. (**C**) Frequency sweep test at varying angular frequency. (**D**) Temperature ramp viscosity test from 5 °C to 50 °C. (**E**) Storage (G′) and loss (G′′) moduli as a function of temperature to evaluate gelation point. (**F**) Alternate step strain test at varying strain of 0.1% or 100%. (**G**) Viscosity versus shear rate (1/s) at 25 °C. The illustrations were Created in BioRender. Janjic, J. (2026) https://BioRender.com/iz7x6hz.

The developed TAC-NE was incorporated into a thermoresponsive Pluronic F-127-based hydrogel matrix using THINKY or a planetary mixer to formulate TAC-NEG as illustrated in Figure 3A. A comprehensive rheological characterization was performed for TAC-NEG which is a single-nanocarrier-loaded hydrogel where the final concentration of F-127 was 18%. To determine the linear viscoelastic region, an oscillation amplitude test was performed at 0.1% to 100% strain; as shown in Figure 3B, the TAC-NEG maintains its stable linear viscoelastic region up to 10% of oscillation strain and beyond that a decrease in storage modulus (G′) is seen, indicating structural disruption of the gel matrix. The oscillation frequency study in Figure 3C shows that the TAC-NEG maintained a consistent storage modulus across a varying angular frequency ranging from 0.1 to 100 rad/s, indicating stable viscoelastic behavior when the oscillation strain was at 0.1%. The temperature ramp study in Figure 3D showed that the viscosity of TAC-NEG increased with increasing temperature, confirming the thermoresponsive behavior of the hydrogel. In Figure 3E, the TAC-NEG showed an increase in storage modulus (G′) with increasing temperature. The storage modulus G′ exceeded the loss modulus G′′ at nearly 23 °C, indicating the onset of gelation, and at 37 °C the TAC-NEG transitioned into complete gelation with G′ > G′′. To evaluate the injectability profile of TAC-NEG, the alternate step strain test was performed and Figure 3F demonstrates the reversible viscoelastic behavior with recovery of storage modulus from 100% to 0.1% strain. In Figure 3G, the viscosity of TAC-NEG was reduced when shear rate (1/s) was increased at 25 °C, confirming the shear thinning behavior of the gel.

**Figure 4:**
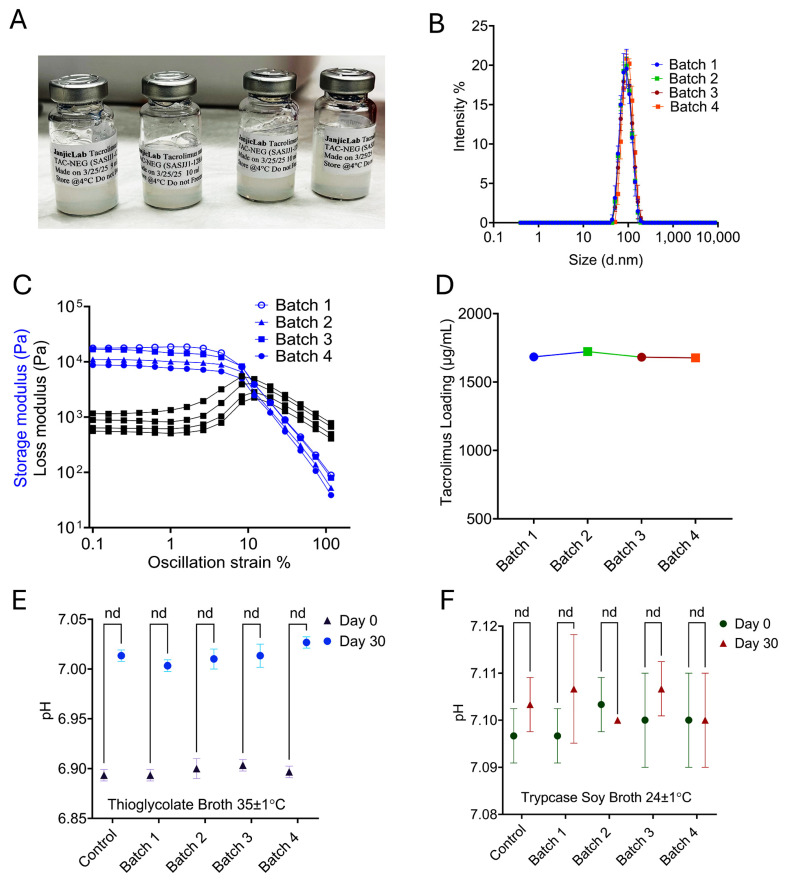
Multiple sterile batch production of TAC-NEGs. (**A**) Image of 4 representative batches of TAC-NEGs. (**B**) Particle size distribution of TAC-NEGs for multiple batches. (**C**) The oscillation amplitude test for multiple batches of TAC-NEG at 0.1% to 100% oscillation strain. (**D**) The tacrolimus encapsulation in µg/mL for multiple batches quantified by using optimized RP-HPLC. Measurement of pH when TAC-NEG batches are in (**E**) thioglycolate medium and (**F**) trypcase soy broth (*n* = 3). Statistical analysis was performed using the Mann–Whitney test, and nd indicates no significant difference (*p* > 0.05).

To support translational development, TAC-NEGs were prepared under aseptic conditions to formulate sterile and reproducible batches intended for advanced preclinical evaluation. Figure 4A shows multiple sterile batches of TAC-NEG prepared under aseptic conditions and filled into injection vials. The droplet distribution for each batch overlapped with each other, indicating similar particle size distribution profile as demonstrated in Figure 4B. The independent batches of TAC-NEG also showed comparable overlapping storage modulus (G′) profiles, indicating consistent and reproducible rheological propertiesas shown in Figure 4C. The tacrolimus concentration across independently prepared TAC-NEG batches showed minimal variation and an average of 1.69 ± 0.2 mg/mL, where the standard deviation represents batch-to-batch variability (Figure 4D). Sterility testing of TAC-NEG batches was performed according to USP 71 [52]. As shown in Figure 4E, no changes in pH were observed over 30 days when the TAC-NEG batches were incubated in thioglycolate medium at 35 ± 1 °C compared to the control medium. Similarly, in Figure 4F, incubation in trypcase soy broth at 24 ± 1 °C showed no significant changes in pH across all batches over the study period. Statistical analysis using the Mann–Whitney test showed no significant differences between groups across all time points (*p* > 0.05), as in Figure 4E,F. These results indicate the absence of microbial contamination, confirming sterile TAC-NEG batches under the tested conditions.

### 3.3. QbD-Driven Optimization of Tacrolimus-Loaded Micelle

#### 3.3.1. The Quality Target Product Profile (QTPP) and Critical Quality Attributes (CQAs)

In the QbD-driven approach for optimization [43,53,54], the QTPP for the TAC-micelle formulation was established first for guiding the development of a stable micelle carrier, suitable for incorporating into the hydrogel matrix. The key goal was to design a micelle formulation with high tacrolimus loading efficiency while maintaining colloidal stability including particle size, polydispersity index (PDI) and drug loading efficiency during storage. The micelle formulation was designed to be in liquid dosage form with a small particle size (15–30 nm) to promote efficient T-cell uptake [33]. Based on the QTPP, the CQAs were defined and specifications were selected. The selected CQAs were particle size, polydispersity index (PDI), drug loading efficiency and %change in colloidal stability and drug loading efficiency after one month of storage [41,55].

As shown in Table 2, the particle size was specified within the 15–30 nm range for targeting T cells enhancing tissue penetration and a PDI < 0.3 for maintaining homogeneity. The tacrolimus loading efficiency in the micelle formulation is specified to be ≥70% to ensure therapeutic efficacy. Since the retention of the drug in the micelle carrier during storage is challenging, the percent change in drug loading efficiency after one month of storage was specified to be ≤5% as a predefined stability criterion, along with particle size and PDI [41,45,46,55]. The method or instrument utilized and the acceptance criteria for each CQAs are mentioned in Table 2.

#### 3.3.2. Full Factorial Design of Experiment for Micelle Formulation Optimization

The objective of screening TAC-micelle formulations with varying concentrations of Pluronic P105 and CrEL was to optimize a micelle carrier that can load TAC. As described in the Materials and Methods Section, a full-factorial DoE was performed where the surfactant concentrations were changed while keeping the TAC concentration constant (0.005% *w*/*w*). The dependent or response factors from the 3^2^ full-factorial DoE runs (Table 1) are presented in Table 3. The summary of the dependent variables showed that initially on Day 1, only two formulations (Run 1 and Run 7) demonstrated a monomodal distribution of particle size with no secondary peak. However, the remaining runs showed bimodal distribution with multiple peaks indicating heterogeneous particle populations. Despite the observed heterogeneity in size distribution, some formulations (Runs 1, 3, 4, and 7) exhibited PDI values below 0.3 in day 1, indicating acceptable uniformity. Several runs also met the predefined specification for initial TAC loading efficiency, achieving a percentage of drug loading efficiency greater than 70%. However, after one month of storage, changes in drug loading efficiency were observed, suggesting that formulation composition influenced tacrolimus retention during storage. Among the nine experimental runs, only Run 1 satisfied all predefined CQA specifications, as shown in Table 3 and highlighted in gray.

#### 3.3.3. Analysis of DoE Runs and Preparation Method for Micelle Formulations

**Figure 5:**
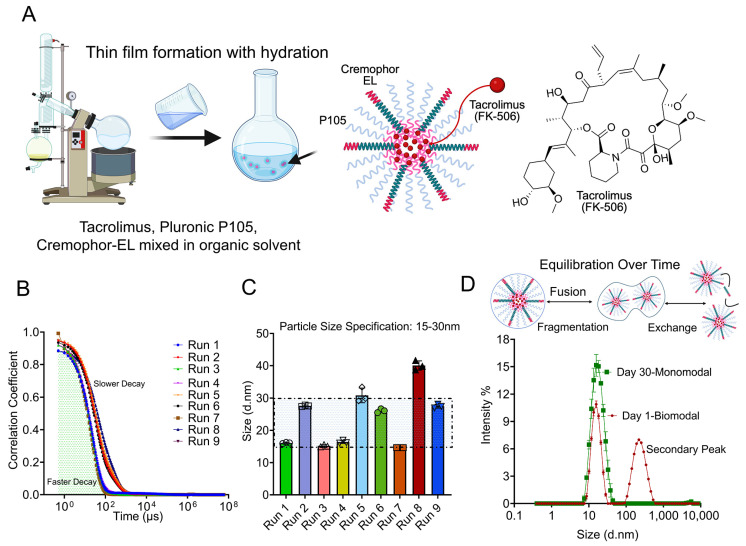
Preparation and characterization of DoE runs. (**A**) Schematic representation of micelle preparation using thin-film hydration method following solvent evaporation and hydration to form self-assembled TAC-micelles. (**B**) Correlation coefficient as a function of time measured by dynamic light scattering (DLS) for all the experimental runs. (**C**) Particle size (nm) of micelle formulations from DoE runs and highlighting the target particle size range. (**D**) Representative particle size distribution of micelle formulations, showing equilibration behavior over time. Data are presented as the mean ± standard deviation (SD) (*n* = 3). The illustrations were Created in BioRender. Janjic, J. (2026) https://BioRender.com/c36p9ne.

TAC-micelles were prepared using a thin-film hydration method, where formulation parameters were varied according to the design of experiments (DoE). The process involved three steps: pre-mixing in an organic solvent, solvent evaporation to form a thin film, and subsequent hydration to produce micellar structures (Figure 5A). As shown in Figure 5B, particle size varied across different DoE runs, indicating formulation impact. Runs 1, 3, and 7 exhibited a faster decay in the correlation function when plotted against time, indicating comparatively smaller particle sizes than the other formulations. The particle size specification range (15–30 nm) is highlighted in Figure 5C, identifying runs that met the desired size criteria, where Run 8 showed the highest particle size (40.06 nm) and Runs 1, 3 and 7 were comparatively smaller (<20 nm). Analysis of particle size distribution showed that some formulations exhibited a bimodal distribution on Day 1, which transitioned to a monomodal distribution by Day 30. This behavior was observed in multiple batches. A representative example (Run 2) is shown in Figure 5D, where the initial bimodal distribution evolved into a monomodal distribution over time. This transition suggests that the micellar system undergoes structural rearrangement during storage, likely due to processes such as fusion, fragmentation, and chain exchange among Pluronic and Cremophor EL components, leading to a more stable micellar population over time [56].

Moreover, the effect of formulation variables on TAC loading was evaluated using statistical analysis. The percentage change in drug loading over 1 month was used as the response variable to assess the stability of the micellar formulation. The percentage change in drug loading was calculated as

(2) $$\frac{\mathrm{Drug}\mathrm{loading}\mathrm{after}1\mathrm{month}-\mathrm{Initial}\mathrm{drug}\mathrm{loading}}{\mathrm{Initial}\mathrm{drug}\mathrm{loading}}\times 100.$$

**Figure 6:**
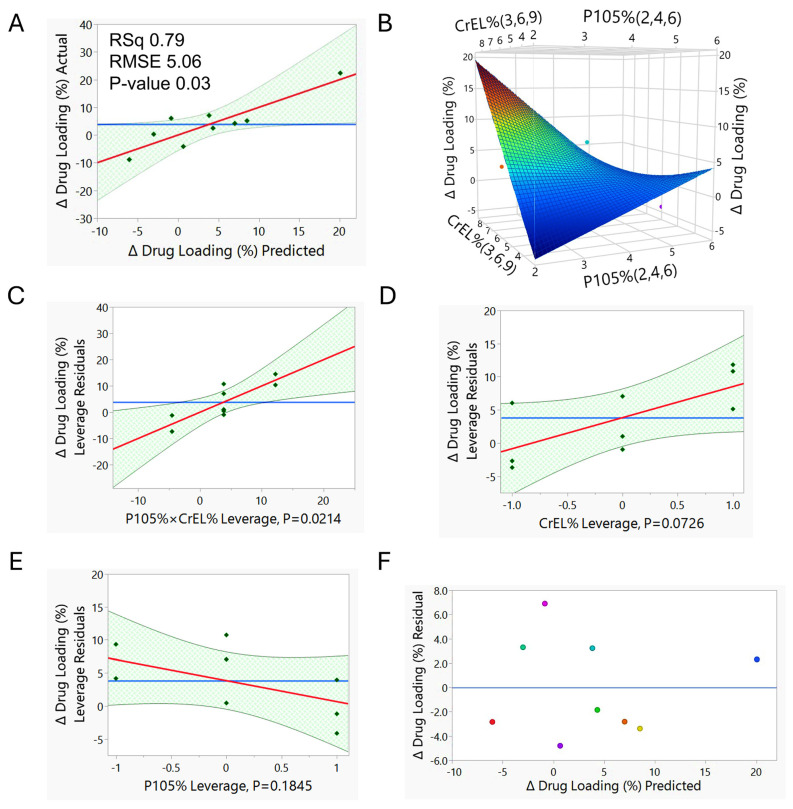
Design of experiments (DoE) modeling and statistical analysis of TAC-micelle formulations. (**A**) Predicted versus actual plot for drug loading (%), demonstrating model fit (R^2^ = 0.79, RMSE = 5.06) and statistical significance (*p* = 0.03). (**B**) The surface plot illustrates the combined effect of surfactants. (**C**–**E**) Leverage plots showing the effects of interaction (CrEL × P105) and individual factors (CrEL and P105) on drug loading, indicating a significant interaction effect (*p* < 0.05) and non-significant individual effects (*p* > 0.05). (**F**) Residual plot showing random distribution of residuals.

Multiple linear regression analysis was performed to evaluate the contribution of individual factors and their interaction. The actual versus predicted plot in Figure 6A showed an R^2^ value of 0.79 and a statistically significant model with a *p*-value of 0.03. The response surface plot in Figure 6B demonstrated a curvature across the design space, indicating a combined effect of Cremophor EL (CrEL) and Pluronic P105 on %change in drug loading. Additionally, the leverage plots confirmed that the combined effect of CrEL and P105 was statistically significant, as indicated by the interaction term (CrEL × P105, *p* < 0.05) shown in Figure 6C. In contrast, the individual effects of CrEL and P105 were not statistically significant (*p* > 0.05), suggesting that drug loading is primarily influenced by their interaction rather than independent contributions (Figure 6D,E). In Figure 6F, the residual analysis showed random distribution of data points without any observable pattern, indicating that the samples were randomly distributed without any pattern. Overall, the DoE analysis highlights that the interaction between surfactants plays a significant factor in TAC retention in the micellar system.

#### 3.3.4. Optimized TAC-Micelle Formulation

**Figure 7:**
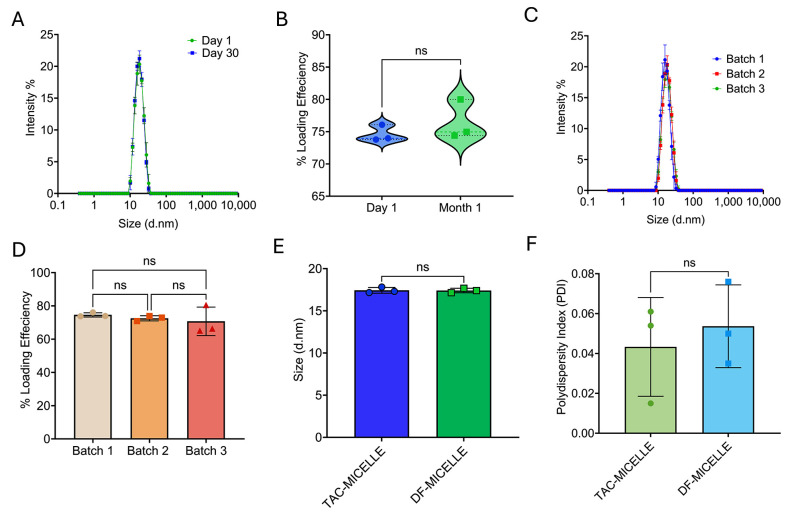
Optimized TAC-micelle formulation. (**A**) Particle size distribution of TAC-micelle from Day 1 to Day 30. (**B**) Percentage drug loading efficiency of TAC-micelle at Day 1 and Month 1. (**C**) Particle size distribution of replicate TAC-micelle batches. (**D**) Percentage drug loading efficiency across three TAC-micelle batches. (**E**) Particle size comparison between DF-micelle and TAC-micelle. (**F**) Polydispersity index (PDI) comparison between DF-micelle and TAC-micelle. Data are presented as the mean ± standard deviation (SD) (*n* = 3), with ns denoting non-significant.

Following the analysis of DoE runs, the micelle formulation was selected. The optimized TAC-micelle formulation exhibited a stable, monomodal particle size distribution over 30 days as shown in Figure 7A. The percentage loading efficiency evaluated on Day 1 and Month 1 showed no significant difference, as shown in Figure 7B. Replicate batches of TAC-micelles demonstrated overlapping particle size distribution profiles (Figure 7C), indicating the reproducibility of the formulation. Consistent with this, the percentage loading efficiency across batches remained comparable without significant variation, as shown in Figure 7D. Furthermore, comparison between DF-micelle and TAC-micelle showed similar particle size and PDI, with no significant differences observed (Figure 7E,F), indicating that drug incorporation did not impact micelle size or dispersity.

### 3.4. Production and Rheological Characteristics of TAC-Dual HG

**Figure 8:**
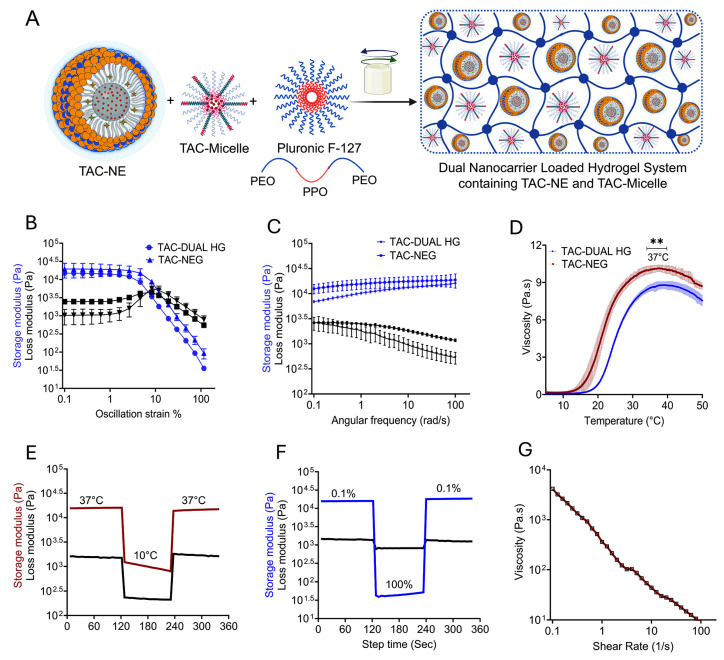
Formulation and rheological characteristics of tacrolimus-loaded dual nanocarrier incorporated hydrogel. (**A**) Schematic illustration of TAC-NE and TAC-micelle incorporation into Pluronic F-127-based hydrogel using planetary or THINKY mixer. Comparison of TAC-NEG and TAC-Dual HG. (**B**) Oscillation amplitude test, (**C**) oscillation frequency test and (**D**) temperature ramp up test. Statistical analysis was done using an unpaired *t*-test with ** denoting *p*-value < 0.05. (**E**,**F**) Alternate step strain test at varying temperature 37 °C and 10 °C and strain at 0.1% or 100% for TAC-Dual HG. (**G**) Viscosity versus shear rate (1/s) at 25 °C of TAC-Dual HG. The illustrations were Created in BioRender. Janjic, J. (2026) https://BioRender.com/x13w4kf.

Following the optimization of two nanocarriers with distinct particle sizes around 81.29 nm ± 1.94 for TAC-NE and around 17.43 nm ± 0.34 for TAC-micelle, they were dispersed into the three-dimensional network of Pluornic-F127-based hydrogel as illustrated in Figure 8A. The final concentration of 18% F-127 was maintained as TAC-NEG. However, the volume of TAC-NE was adjusted to incorporate TAC-micelle. The final tacrolimus concentration of this dual hydrogel was 1.21 ± 0.03 mg, and around 98% tacrolimus was encapsulated into the hydrogel matrix. The rheological characterization of TAC-Dual HG was performed and compared with TAC-NEG. In Figure 8B, the oscillation amplitude and frequency test showed that similar to TAC-NEG, the dual-HG also maintained a linear viscoelastic region and a consistent gel behavior with varying angular frequency (rad/s). In Figure 8B,C, the oscillation amplitude and frequency sweeps showed that, like TAC-NEG, the dual nanocarrier hydrogel maintained a linear viscoelastic region and exhibited consistent gel behavior across the tested angular frequency range of 0.1–100 rad/s. The temperature ramp up study showed that the viscosity of TAC-NEG was comparatively higher than that of TAC-Dual HG. At 37 °C, the viscosities of the two formulations were significantly different, confirmed by an unpaired *t*-test with a *p*-value < 0.005 (Figure 8D). In Figure 8E,F, the thermoreversibility and injectability of the dual nanocarrier hydrogel were evaluated. The TAC-Dual HG showed reversible behavior, with recovery of the storage modulus (G′) upon transitioning from low-to-high temperature (10–37 °C) and from high-to-low strain (100% to 0.1%), indicating preservation of gel structure under varying conditions. Furthermore, the viscosity vs. shear rate (1/s) profile showed decreased viscosity with increasing shear rate, confirming shear-thinning behavior of the hydrogel (Figure 8G).

### 3.5. In Vitro Release Evaluation Using Dissolution and Franz Diffusion System

**Figure 9:**
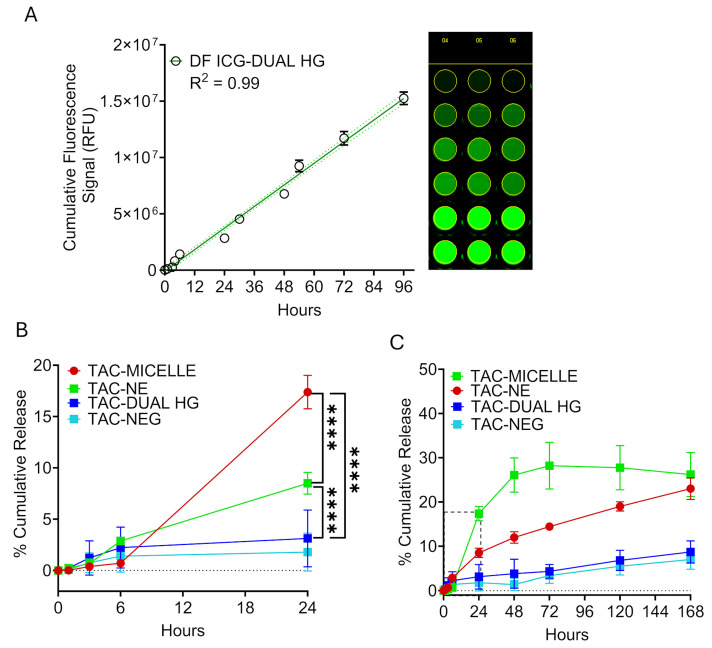
ICG-labeled DF-NE release from DF-DUAL HG and tacrolimus release from nanoformulations. (**A**) The ICG-DFNE release study from the DUAL HG matrix using the dissolution method. (**B**) Percentage cumulative release of tacrolimus (normalized to loaded dose) from TAC-micelle, TAC-NE, TAC-DUAL HG, and TAC-NEG over 24 h. (**C**) %Cumulative release of loaded tacrolimus dose until 168 h. Data are presented as the mean ± standard deviation (*n* = 3). Statistical analysis was performed using two-way ANOVA followed by Tukey’s multiple comparison test; **** *p* < 0.0001.

For monitoring the release of NE droplets from the hydrogel matrix in the dual system, the fluorescence release was evaluated using the dissolution method. The fluorescence signal was measured at different time intervals, and an increase in cumulative fluorescence signal was observed until 96 h as shown in Figure 9A. A simple linear regression analysis was performed, and it showed a linear relationship between cumulative fluorescence signal and time (R^2^ = 0.99), indicating a consistent release profile of the ICG-labeled nanoemulsion from the dual hydrogel system. Moreover, the TAC diffusion across nanocarriers was evaluated using a Franz diffusion system. Figure 9B shows that at 24 h, the cumulative percentage of TAC released (normalized to the loaded dose) differed across the formulation groups. The highest release was observed for TAC-micelle, followed by TAC-NE, while TAC-DUAL HG and TAC-NEG exhibited significantly lower (*p*-value < 0.0001) release compared to these formulations. Figure 9C demonstrates the cumulative TAC release profile until 168 h. The dotted box highlights the differences in release behavior that became apparent after 24 h. The TAC-DUAL HG and TAC-NEG showed a slow and sustained release till 168 h, with cumulative release values of 8.72 ± 2.5% and 7.01 ± 2.01%, respectively, compared to higher release from TAC-micelle (26.21 ± 4.97% and TAC-NE (23.04 ± 2.45%).

**Figure 10:**
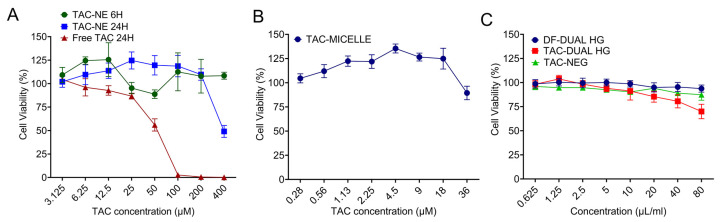
Raw 264.7 macrophage viability following exposure to tacrolimus formulations. (**A**). Macrophage viability determined using an ATP-based CellTiter-Glo^®^ 2.0 assay following exposure to TAC-NE and free tacrolimus for 6 h and 24 h. (**B**). Macrophage viability following 24 h exposure to TAC-micelle. (**C**). Macrophage viability following exposure to drug-free dual hydrogel (DF-Dual HG), TAC-Dual HG, and TAC-NE over a concentration range of 0.625–80 µL/mL. Cell viability is expressed as percentage relative to control. Data are presented as the mean ± SD (*n* = 6 independent cell cultures per group).

Macrophage viability was evaluated using an ATP-based CellTiter-Glo^®^ 2.0 luminescence assay [17]. The tacrolimus concentration differed across formulations, with TAC-NE prepared at a higher drug loading (3.9 mg/mL). Despite this higher concentration, TAC-NE did not reduce cell viability at 6 h and 24 h compared to the Free-TAC solution shown in Figure 10A. TAC-micelles, prepared at lower drug concentrations, also maintained high cell viability as shown in Figure 10B. Due to differences in tacrolimus loading, hydrogel formulations were evaluated based on formulation volume (µL/mL). Both DF and TAC-Dual HG maintained cell viability across the tested concentration range in Figure 10C. Overall, the viability profiles indicate that the formulations are cytocompatible with RAW 264.7 macrophages under the tested conditions.

## 4. Discussion

The results of this study demonstrated the prospects of developing a thermoresponsive dual nanocarrier hydrogel system with the goal of local TAC delivery. The preliminary analysis of the individual nanocarriers indicated that both systems could be developed with physicochemical properties suitable for successful incorporation into the hydrogel matrix. The development of a novel nanoemulsion carrier with a higher dose of TAC loading was driven by the previously reported parameters of the TAC-loaded nanoformulation [17,41]. The LM20 microfluidization at 20,000 PSI effectively processed and reduced the particle size within the 80 nm range (Figure 1). The initial concept regarding the solubility of TAC in coconut oil, Pluronic P105, and CrEL was derived from the existing literature [17,41,57]. Nonetheless, the solubility characteristics of TAC at temperatures exceeding 37 °C were evaluated with consideration of the manufacturing processing temperature, which indicated that TAC exhibits enhanced solubilization in the surfactants and oil phase for an improved encapsulation profile of TAC in the nanoformulations (Figure 2). Ensuring reproducibility while controlling temperature during production presents challenges in formulation development. We have employed a reliable, robust and reproducible processing technique validated through the production of three independent batches. The process optimization is critical for coconut oil-based colloidal systems as they show sensitivity towards formulation composition and processing conditions [58]. A noteworthy advancement of this study is the effective integration of tacrolimus at a relatively high loading level in the NE, while still achieving around 80% drug loading efficiency. This is especially significant due to the lipophilic nature of tacrolimus, which makes it challenging to formulate at higher concentrations [59]. Temperature sensitivity poses a significant challenge for formulations loaded with TAC. Following ICH guidelines, assessments were conducted at various temperatures, ultimately leading to the selection of NE for storage at 4 °C [60]. Furthermore, the clinically approved fluorescent dye ICG was successfully integrated into this nanoemulsion, highlighting its potential to function as a theranostic nanocarrier for future clinical translational work [17,28]. Our previous study with TAC-NE showed efficient uptake of Raw 264.7 macrophages resulting in decreased inflammatory cytokine levels [17]. Both T-cells and macrophages play a role in the overexpression of immune cells that contribute to a self-amplifying cytokine loop [61,62,63]. To broaden this approach beyond macrophage-targeted drug delivery, an additional nanocarrier system was incorporated into the platform. Micelles as the second nanocarrier system were developed for loading TAC to support targeting T-cells [33]. In comparison to nanoemulsion, they were designed with smaller particle size to facilitate faster TAC release [64]. The optimization was driven by the QbD approach which was initiated with the identification of QTPPs and CQAs along with the objective being focused on immune cell targeting and emphasizing the retention capacity of tacrolimus.

Our earlier research reported an optimized micelle formulation utilizing Pluronic P105 and CrEL for loading hydrophilic drugs [45,46,65]. Building upon this foundation, we varied the ratios and concentrations of these surfactants to systematically evaluate the impact and optimize TAC loading and retention in micelles in this study (Table 1). Additionally, the equilibration of the micellar formulation has been observed over time in specific experimental trials (Table 3). Pluronic P105, containing long polymer chains of PPO-PEO-PPO when combined with the amphiphilic molecule CrEL, undergoes fusion, fragmentation, and exchange processes as it equilibrates over time [56,66]. As a result, the bimodal distribution that was observed following the initial micelle formation eventually transitioned to a monomodal distribution when evaluated after one month (Figure 5). The statistical analysis of the results on TAC retention showed that the surfactant interactions play a critical role for retaining the percentage of loading efficiency within one month. This highlights the process of self-assembling CrEL and P105 to form the micelle core, effectively retaining the drug [67]. TAC retention within the micelle core is challenging. However, after successful evaluation of DoE experimental results, TAC-micelle was selected with enhanced TAC retention capacity and particle size ranging from 15 to 17 nm and percentage of loading efficiency > 72% (Figure 7).

The distinct nanocarriers including nanoemulsion and micelles were developed with the goal of developing a localized drug delivery platform. Due to delivery constraints and frequent systemic adverse effects, TAC, being a potent immunosuppressant, must be administered locally [59]. To address that, a local depot platform was first established incorporating a single nanocarrier-loaded hydrogel (TAC-NEG) keeping 18% of F-127 as the final concentration [17,45]. Sterile batches of TAC-NEGs were produced, confirming reproducible profiles (Figure 4). In this work, we developed a novel dual nanocarrier-based hydrogel platform where both nanoemulsion and micelle carriers were dispersed into a thermoresponsive Pluronic F-127-based hydrogel (Figure 8). The TAC Dual HG exhibited thermoresponsive behavior forming a complete gel at 37 °C, which supports in situ gelation upon administration. The rheological characteristics were evaluated and compared with single nanocarrier-loaded hydrogel (TAC-NEG). The shear thinning behavior of Dual-HG and the alternate step strain study confirmed its suitability for injection [45]. The percentages of nanoemulsion and micelles were adjusted so that both single and dual hydrogel contained the same final concentration of Pluronic F-127 (18%), which is critical for gelation [17,46,68]. Notably, the hydrogels were developed at a 25 mL batch scale, indicating a relatively higher formulation development scale, and showed an excellent in vitro viability profile (Figure 10) that may be relevant for further translational work. The clinically approved ICG dye [69] was labeled in NE in DF Dual-HG which enabled tracking of NE droplets released from the hydrogel matrix. However, fluorescent-labeled micelles were not included because stable fluorescent dye incorporation in micellar systems require separate formulation optimization [70] The in vitro drug-release testing demonstrated the following tacrolimus release profile: TAC-micelle > TAC-NE > TAC-Dual HG > TAC-NEG when tested in a Franz diffusion system (Figure 9). The faster release profile from TAC-micelle is related to its reduced particle size and the increased surface area that facilitates drug diffusion [71], in comparison to TAC-NE. However, the integration of the nanocarriers within the hydrogel matrix resulted in a slow and sustained TAC release profile. As at 37 °C, the formulations form a three-dimensional gel network [17,45,68], limiting nanocarrier diffusion and ultimately slowing the TAC release profile. In the Dual-HG, the micelle represented only one third of the NE fraction to avoid any burst release. For that, their limited proportion did not impact significantly on cumulative TAC release compared to TAC-NEG. The lower cumulative release from the hydrogels is expected and supports the design rationale for localized and prolonged TAC delivery.

Notably, the study highlights that engineering particle size could be used as a strategy to modulate release behavior that may support differential interaction with relevant immune cell populations [33,34]. The advantage of including smaller micellar carriers is that they may diffuse earlier from the matrix and potentially contribute to earlier modulation of T-cell-mediated immune signaling, while the nanoemulsion may provide a complementary pharmacological effect through targeting macrophages [17,72]. However, the regenerated cellulose membrane-based results are interpreted as formulation-controlled in vitro release or diffusion behavior of tacrolimus. In vivo TAC release from hydrogel release would require further evaluation as it is governed by additional factors including the local inflammatory microenvironment and tissue responses around the depot [73]. Thus, while this study is limited to formulation platform development with engineered nanocarriers, the in situ release and local immunomodulatory effects require further biological evaluation in relevant in vitro and in vivo models.

## 5. Conclusions

Therefore, this work demonstrated successful production of a sterile thermoresponsive hydrogel platform loaded with dual nanocarriers for localized immunosuppression through TAC delivery. The incorporation of two independently developed distinct nanocarriers, including NE and micelles, into the hydrogel resulted in desirable in situ gelation attributes, colloidal stability, and extended drug-release behavior. The distinct particle size-dependent release behavior of the nanocarriers could be used as a platform for modulating the release behavior. The findings showed that this dual nanocarrier strategy could provide a promising approach for forming a localized tacrolimus depot and supporting prolonged drug release. Sterile hydrogel batch production (25 mL scale) and biocompatibility further support the ongoing assessment of this platform for future preclinical and translational studies. Our future work will focus on further validation of this TAC Dual-HG under relevant in vitro and in vivo conditions.

## Figures and Tables

**Table 1:** Three-level, two-factor full-factorial design of experiments (DoE) used for formulation optimization. The independent variables were Pluronic P105 and Cremophor EL (CrEL), evaluated at three coded levels: −1 (low), 0 (center), and +1 (high).

Run	P105 (Coded)	CrEL (Coded)	P105 (%)	CrEL (%)
1	+1	−1	6	3
2	+1	0	6	6
3	−1	+1	2	9
4	−1	0	2	6
5	0	0	4	6
6	+1	+1	6	9
7	−1	−1	2	3
8	0	−1	4	3
9	0	+1	4	9

**Table 2:** Critical quality attributes (CQAs) and predefined specifications for TAC-micelle developed using a QbD approach.

Critical Quality Attribute (CQA)	Specification	Measurement Method	Acceptance Criteria	Justification
Particle Size (nm)	15–30 nm	DLS	Within target range with narrow particle size distribution	Ensures efficient cellular uptake (e.g., T cells and macrophages) and enhanced tissue penetration for localized delivery.
Polydispersity Index (PDI)	<0.3	DLS	≤0.3	Indicates uniform size distribution, ensuring formulation homogeneity and reproducibility.
Drug Loading Efficiency (%)	>70%	RP-HPLC	>70%	For adequate drug incorporation to achieve therapeutic efficacy.
Particle Size after 1 Month	15–30 nm	DLS	≤10% change in particle size	Confirm physical stability during storage.
Polydispersity Index (PDI) after 1 Month	<0.3	DLS	≤10% change in PDI	Maintains uniformity and prevents aggregation over time.
% Change in Drug Loading Efficiency after 1 Month	≤5%	RP-HPLC	≤5% change in drug loading	Indicates drug retention within the carrier system and ensures chemical and physical stability during storage.

**Table 3:** Summary of dependent responses obtained from the 3^2^ full-factorial design of experiments (DoE) for micellar formulation optimization.

Run	Dependent Factors										
	Initial Distribution Type	Particle Size (nm)			Polydispersity Index (PDI)	Initial Drug Loading Efficiency %	Particle Size After 1 Month (nm)	PDI After 1 Month	Distribution Type After 1 Month	Drug Loading Efficiency After 1 Month	% Δ Drug Loading
		Peak 1	Peak 2	Peak 3							
1	Monomodal	16.7	x	x	0.19	74.6	15.9	0.05	Monomodal	76.45	2.48
2	Bimodal	16.2	241.2	x	0.66	62.6	16.4	0.14	Monomodal	60	−4.14
3	Bimodal	15.5	3854	x	0.22	61.7	15.2	0.28	Monomodal	75.5	22.36
4	Bimodal	15.9	1161.3	1594.7	0.27	68.9	16.3	0.34	Monomodal	71.8	4.2
5	Bimodal	83.7	136.9	x	0.69	70.8	14.4	0.08	Monomodal	75.8	7.06
6	Bimodal	15.8	241.5	x	0.63	70.5	15.2	0.11	Monomodal	70.71	0.29
7	Monomodal	15.8	x	x	0.09	79	15.6	0.14	Monomodal	72	−8.86
8	Bimodal	209.3	17.0	1589.66	0.91	59.5	15.9	0.07	Monomodal	63.1	6.05
9	Bimodal	15.3	229	x	0.68	73.8	14.3	0.06	Monomodal	77.6	5.14

## Data Availability

Data can be made available for non-commercial use upon reasonable request to the corresponding author at janjicj@duq.edu.

## Abbreviations

ICH: International Council for Harmonization; VCA: vascularized composite allotransplantation, TAC: tacrolimus; NE: nanoemulsion; ICG: indocyanine green; NEG: nanoemulgel, HG: hydrogel.
